# A quantitative proteomics analysis for small molecule Stemazole’s effect on human neural stem cells

**DOI:** 10.1186/s12953-020-00168-2

**Published:** 2020-12-09

**Authors:** Huajun Li, Yubo Zhang, Jing Zhang, Chaoran Zhao, Yizi Zhu, Mei Han

**Affiliations:** Key Laboratory of Radiopharmaceuticals, Ministry of Education, College of Chemistry, Beijing Normal University, Beijing, 100875 China

**Keywords:** Stemazole, Small molecule, Neural stem cells, Proteomics, Protein expression profiling

## Abstract

**Background:**

Stemazole is a novel small molecule that has been suggested to have the ability to protect multiple stem cells. The proliferation-promoting activity and promising neuroprotective effects of stemazole make it a prospective drug for neurodegenerative disease treatment.

**Methods:**

Since previous studies have shown that it protective effect in extreme conditions, to understand more aspects of stemazole, in this study, a systematic tandem mass tags (TMT)-labelled proteomics approach was used to address the whole proteome expression profile with or without stemazole in normal conditions instead of extreme conditions. Bioinformatics analyses, including Gene Ontology (GO), Kyoto Encyclopedia of Genes and Genomes (KEGG) pathway enrichment and protein-protein interaction (PPI) network analyses, were employed.

**Results:**

The effect of stemazole on the expression profiles of neural stem cells was obtained. A total of 408 proteins with changes at the abundance level of two groups were identified: 178 proteins increase in abundance and 240 proteins decrease in abundance, respectively. Low abundance of some mitochondrial respiratory chain enzyme, overproduction of reactive oxygen species (ROS) and reduction of mitochondrial membrane potential may indicate stemazole has cytotoxicity.

**Conclusions:**

It is the first proteomics research about stemazole, and the possible cytotoxicity of stemazole has been reported for the first time. The information about proteins that were affected by stemazole and more characteristics of stemazole will help obtain a complete picture of this small molecule drug. These findings provide a scientific basis for further stemazole treatment research.

**Supplementary Information:**

The online version contains supplementary material available at 10.1186/s12953-020-00168-2.

## Background

Currently, the problem of global ageing is serious. The incidence and mortality rates of neurodegenerative diseases have risen sharply; Alzheimer’s disease (AD) and Parkinson’s disease (PD) are two common progressive central nervous system degenerative diseases in the elderly [1–3]. Unfortunately, the aetiologies of these diseases are unknown, and their pathology is unclear; moreover, the drugs that have been found to treat these diseases are extremely rare and can only relieve the symptoms to a certain extent [4]. In fact, neurodegenerative diseases are caused by damage, degeneration or loss of functional cells. Starting at the root cause of the disease, the use of stem cell drugs to promote the repair potential of stem cells and restore their biological functions seems to be a more ideal treatment [5–8]. Thus, stem cell drug regulatory therapy has become a new strategy for treating neurodegenerative diseases [9, 10]. Small molecules that regulate the activity of stem cells are rare, especially those that have a therapeutic effect on diseases.

The small molecule compound stemazole is a potent stem cell drug that was obtained through high-throughput chemical screening of approximately 25,000 compounds. Stemazole has been found to possess great potential as a promising stem cell drug due to its prominent proliferation-promoting activity in multiple types of stem cells, such as human embryonic stem cells and human adult stem cells, including hippocampal neural/pancreatic/cardiac stem cells [11]. Stemazole can significantly enhance embryonic stem cell survival and prevents apoptosis due to disaggregation or starvation. In addition, stemazole supports long-term stem cell maintenance without compromising self-renewal and pluripotency [12]. For human hippocampal neural stem cells, stemazole exhibits the ability to promote proliferation in a dose-dependent manner and has been found to be a novel and prospective proliferative activator for stem cells [11].

Stemazole has a protective effect on a variety of types of stem cells, especially neural stem cells, and exhibits potential pharmacological activity against nerve injury disease. Previous experimental evidence also reveals stemazole’s neuroprotective effects in vivo. Stemazole demonstrates the effect of improving the memory impairment and neuronal damage in an AD rat model and a PD mouse model. In an Aβ1–40 aggregate injection rat model, stemazole reverses learning and memory deficits, reducing the loss of neurons and degeneration in the hippocampus [13]. In a PD disease model, stemazole has a therapeutic effect against 1-methyl-4-phenyl-1,2,3,6-tetrahydropyridine (MPTP)-induced nerve damage and could play a vital role in behavioural improvement [14]. Demonstrated neuroregenerative effects suggest stemazole is a potential candidate with pharmacological activity that can be used as a therapeutic agent against neurodegenerative diseases.

The pharmacokinetic property of stemazole shows that the small molecule is suitable for oral medication and has low cardiotoxicity; moreover, it is able to cross the blood-brain barrier and stably accumulate [15]. Research on the direct targets and the underlying mechanisms of stemazole through network pharmacology was conducted. The five critical targets were identified, including caspase-3 (CASP3), caspase-8 (CASP8), RAC-alpha serine/threonine-protein kinase (AKT1), mitogen-activated protein kinase 8 (MAPK8) and mitogen-activated protein kinase 14 (MAPK14), and the mitogen-activated protein kinase (MAPK) signalling pathway was eventually screened out [16].

Proteins are the executors of life activities; thus, the qualitative description or quantitative detection of proteins is of great importance. Studies on protein expression and activity with conventional molecular biological approaches can be conducted only on limited numbers of proteins and on a protein-by-protein basis. Unlike conventional molecular biology methods, proteomics has the ability to quantify thousands of cellular proteins comprehensively and accurately [17–19]. To have a more comprehensive understanding of stemazole, in this study, we focused on the effect of stemazole under normal conditions and attempted to assess the effect of stemazole on the protein expression profile of human neural stem cells. Tandem mass tags (TMT)-labelled proteomics, a relative and absolute quantitative technology, was selected to explore the possible impact of stemazole on protein production.

We performed TMT-based quantitative proteome analysis to obtain the protein expression profile of neural stem cells affected by stemazole. Proteins with altered expression in response to stemazole were identified. The protein expression profile from proteomics analysis will provide insight into the global responses of human neural stem cells to stemazole and will offer suitable references and more information for broadening our understanding of the stem cell drug stemazole.

## Methods

### Preparation of the stemazole solution

Stemazole, with a purity > 98%, was synthesized by the Key Laboratory of Radiopharmaceuticals of Beijing Normal University (Beijing, China). The stemazole powder was dissolved in DMEM/F12 medium (Sigma, San Francisco, CA, USA). Gradient dilution was performed to reach the target concentration.

### Cell culture and treatment

Human neural stem cells were obtained from the Stem Cell Centre of Peking University (Beijing, China). The cells were cultured in DMEM/F12 medium with 0.02% fibroblast growth factor (FGF), 0.02% epidermal growth factor (EGF) and 2% B-27 supplements (Gibco, Carlsbad, CA, USA). Neural stem cells were cultured in a 75-cm^2^ culture bottle (cell density was approximately 1 × 10^6^/mL) under the condition of 37 °C and 5% CO_2_. Human neural stem cells in the logarithmic growth stage were rinsed three times with DMEM/F12.

### Protein sample preparation

According to previous experimental results stemazole at 40 μM was the most efficient concentration [11]. 4 days treatment was chosen according the results shown in Additional file 1: Fig. S1. Thus, this concentration was also selected for the exploration of the effect on the expression of cellular proteins. The cells in the stemazole and control groups were inoculated in 6-well plates with or without 40 μM stemazole for 4 days. Each group has three biological independent replicates. Suspension neural stem cells were collected by centrifugation at 1000 g for 5 min. Then, the precipitate was washed with pre-cooled PBS solution (Baoruijie, Beijing, China) 3 times after removing the supernatant. The cells were collected in a 1.5-mL centrifuge tube. The samples were frozen in liquid nitrogen and stored at − 80 °C for later use.

### Total protein extraction

The sample cells were ground into powder in liquid nitrogen and rapidly transferred to pre-cooled centrifuge tubes. Then, samples were mixed with protein lysis buffer containing 100 mM NH_4_HCO_3_, 6 M urea and 0.2% SDS (pH 8). Then, 5 min of ultrasonication on ice for lysis was carried out. After centrifugation at 12,000 x g for 15 min at 4 °C, 10 mM DTT was added for 1 h at 56 °C. The samples were alkylated with sufficient iodoacetamide for 1 h at room temperature and kept away from light. Four times volume pre-cooled acetone was added to precipitate at least 2 h under the condition of − 20 °C. Then, after centrifugation at 12,000 x g for 15 min at 4 °C, the precipitate was collected. Protein dissolution buffer containing 100 mM triethylammonium bicarbonate (TEAB) and 6 M urea (pH 8.5, Baoruijie, Beijing, China) was added to dissolve the protein precipitate.

### Protein quality test

A Bradford protein quantitative kit was used to prepare BSA standard protein solution according to the instructions (Baoruijie, Beijing, China); the gradient concentration ranged from 0 to 0.5 g/L. The protein concentration of the sample was tested according to the manufacturer’s instructions. Each sample of 20 μg of protein was loaded for 12% SDS-PAGE (the stacking gel was run at 80 V for 20 min, and the separation gel was run at 120 V for 90 min). After gel electrophoresis, Coomassie brilliant blue R-250 staining was performed, and the gel destained until the bands were visualized clearly.

### TMT labelling of peptides

To each 120-μg protein sample was added lysis buffer to the total volume of 100 μL, 3 μL of 1 μg/μL trypsin and 500 μL of 50 mM TEAB buffer, and the samples were digested overnight. Then, 1% formic acid was added at an equal volume to the digested sample. The samples were then centrifuged at 12,000 x g for 5 min at room temperature. The supernatant was loaded to a C18 desalination column slowly and washed 3 times with 1 mL of washing solution containing 0.1% formic acid and 4% acetonitrile. Next, 0.4 mL of elution buffer containing 0.1% formic acid and 75% acetonitrile was added for two consecutive elutions. The eluent samples were combined and lyophilized. Then, 100 μL of 0.1 M TEAB buffer was added to reconstitute the sample, 41 μL of acetonitrile-dissolved TMT labelling reagent was added, and the samples were reacted for 2 h. After that, the reaction was terminated by adding 8% ammonia (Baoruijie, Beijing, China). An equal volume of each labelled sample was mixed, desalted and lyophilized. The tag used of stemaozle group was 126.12772, 127.12476, 128.12811 and control group was 129.13147, 130.13482, 131.13818, respectively.

### Separation of fractions

Gradient elution was performed using mobile phases A (2% acetonitrile, pH 10.0) and B (98% acetonitrile, pH 10.0). The lyophilized powder was dissolved in solution A and centrifuged at 12,000 x g for 10 min at room temperature. A C18 column (Waters BEH C18 4.6 × 250 mm, 5 μm) was used. The specific elution gradient is shown in Table 1. The eluates were collected per minute and combined into 10 fractions, which were then lyophilized and reconstituted with 0.1% formic acid.

**Table 1:** Elution gradient table of liquid chromatography for separation of peptide fractions

Time (min)	Flow rate (mL/min)	Mobile phase A	Mobile phase B
0	1	97%	3%
10	1	95%	5%
40	1	80%	20%
68	1	60%	40%
70	1	30%	70%
74	1	0	100%
90	1	0	0

### LC-MS/MS analysis

The mobile phases were prepared. Phases A and B contained 0.1% formic acid in water and 0.1% formic acid in acetonitrile, respectively. An EASY-nLC™ 1200 UHPLC system and a Q Exactive HF-X mass spectrometer (Thermo Fisher Scientific, Waltham, MA, USA) were used. Sample supernatant was injected with 1 μg for detection. The gradient programme is shown in Table 2. An ESI ion source was used for analysing separated peptides; the spray voltage was set at 2.3 kV, and the ion transport capillary temperature was set at 320 °C. The full scan with a resolution of 60,000 (m/z 200) ranged from m/z 350 to 1500. The top 40 highest abundant parent ions were selected. They were fragmented by the higher-energy collisional dissociation (HCD) method and analysed in a secondary mass spectrometry detection (resolution was 45,000 for 10 plex, m/z 200). The raw data for mass spectrometry detection were generated.

**Table 2:** Liquid chromatography elution gradient table

Time (min)	Flow rate (mL/min)	Mobile phase A	Mobile phase B
0	600	94	6
2	600	83	17
82	600	60	40
84	600	45	55
85	600	0	100
90	600	0	100

### Protein identification and quantitation

The spectrum results of each fraction were searched in the Uniprot Human Complete Proteome database using Proteome Discoverer 2.2 (Thermo Fisher Scientific). The mass tolerance search parameters for precursor ions were set at 10 ppm, and the mass tolerance for product ions was set at 0.02 Da. In Proteome Discoverer 2.2, carbamidomethyl was specified as a fixed modification. TMT modification on peptide N-terminus, tyrosine and lysine, oxidation of methionine were specified as variable modifications. A maximum of 2 miscleavage sites were allowed. The identified proteins contain at least one unique peptide with an FDR of no more than 1.0%. Proteins containing similar peptides that could not be distinguished were identified as the same protein group. Reporter quantification of TMT 10-plex was used. The results were statistically analysed using the Mann-Whitney Test, and quantitative proteins with significant differences between stemazole and control groups were defined as differentially abundant proteins.

### Bioinformatics analysis

Analysis of Gene Ontology (GO) (http://www.geneontology.org/) was carried out to catalogue the molecular functions (MFs), cellular components (CCs), and biological processes (BPs). GO annotation was performed to analyse the identified proteins using the interproscan-5 program. GO enrichment was performed using the quantified proteins as background. The enrichment analysis method of the Kyoto Encyclopedia of Genes and Genomes (KEGG) pathway was the same as that in the GO analysis and was used to analyse the protein families and major biochemical metabolic and signal transduction pathways involving the differentially abundant proteins. The protein-protein interaction (PPI) network was analyzed with STRING database and the differentially abundant proteins were visualized with Cytoscape.

## Results

### Proteins identification

**Fig. 1:**
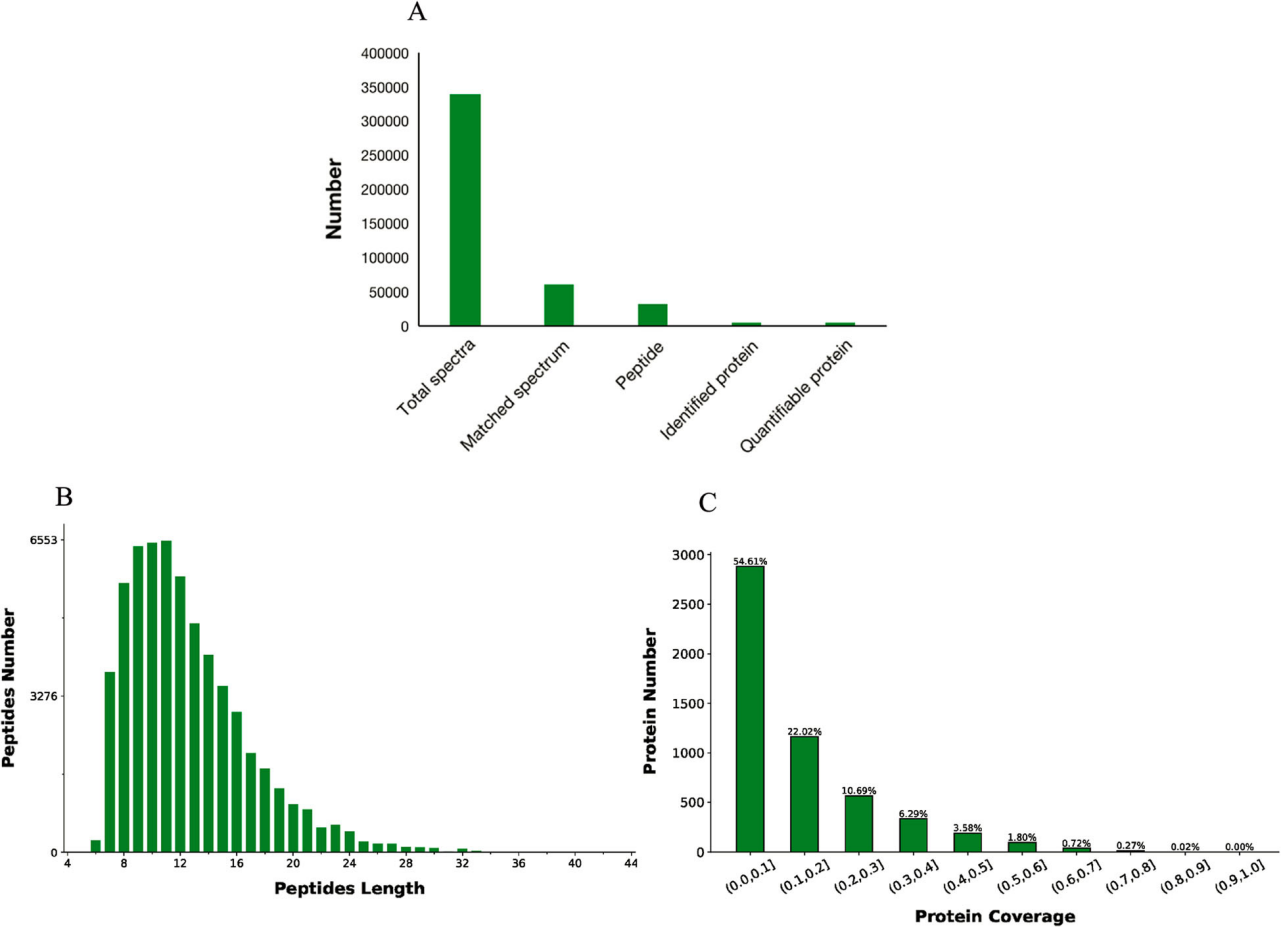
Identification and analysis of proteins. Basic information for protein identification (**a**), peptide length distribution plot (**b**) and coverage of the identified proteins (**c**)

A 10-plex LC-MS/MS analysis was conducted. The raw files were imported into Proteome Discoverer 2.2 software directly for database retrieval and quantification of spectral peptides and proteins. A total of 338,156 spectra were identified from these samples, corresponding to 31,720 unique peptides and 5266 identified proteins, with a false discovery rate (FDR) ≤ 0.01 (Fig. 1a). The peptide length distribution plot and the coverage of the identified proteins were shown in Fig. 1b and c.

### Differentially abundant proteins

**Fig. 2:**
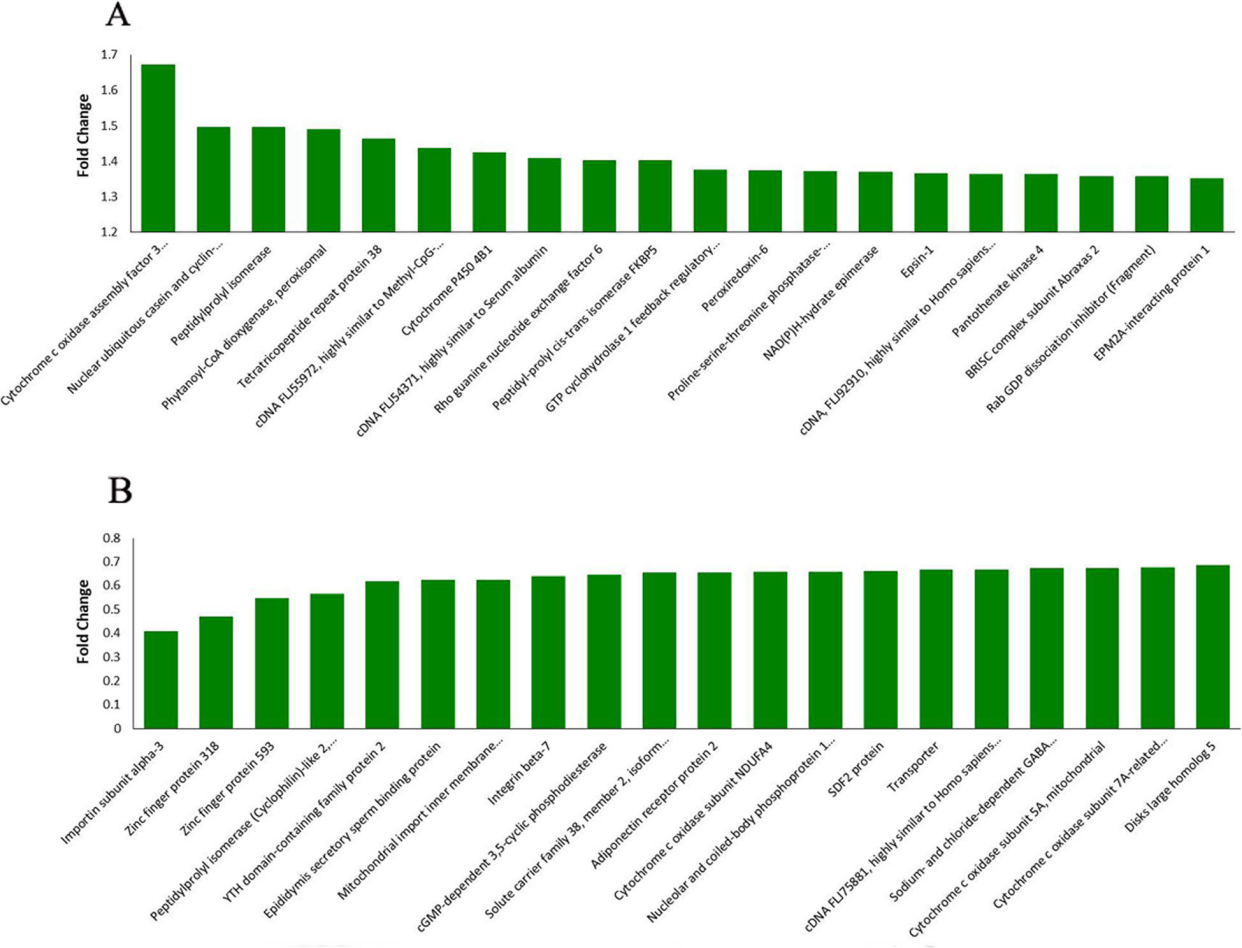
Top 20 up-regulated proteins (**a**) and down-regulated proteins (**b**) in the stemazole group compared to the control

To determine the protein expression profiles affected by stemazole, we used the approach of TMT-labelled proteomics. For protein difference analysis, the ratio of the mean values of all biologically repeated quantitative values of each protein in the control group is taken as a fold change (FC). To determine the significance of the difference, the relative quantitative values of each protein in the two groups were tested by T-test, with *P*-value ≤0.05.When the FC ≥ 1.2 and the P-value ≤0.05, proteins increase in abundance were screened, and when the FC ≤ 0.83 and the P-value ≤0.05, proteins decrease in abundance were selected. Compared with the control group, the stemazole group had 178 proteins with increase in abundance and 230 proteins with decrease in abundance (Additional file 2: Table S1). Among these proteins, the top 20 up- and down-regulated proteins are shown in Fig. 2a and b, respectively.

**Fig. 3:**
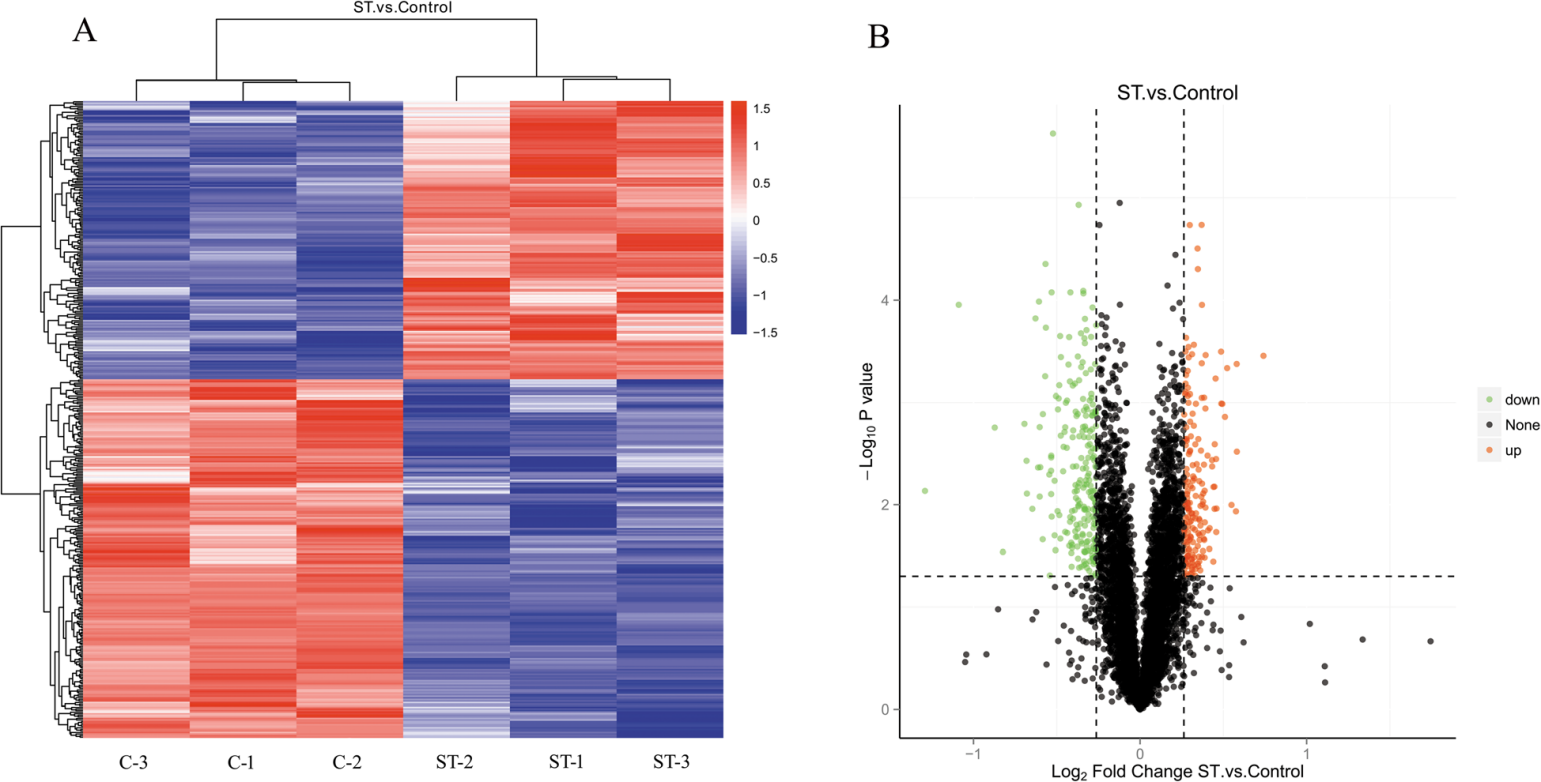
Clustering heat map (**a**) and volcano plots (**b**) of differentially abundant proteins expressed in the stemazole group compared to the control

Cluster analysis was carried out on the relative contents of the different proteins in each sample. The up- and down-regulation of different proteins in three independent replicates of each group were observed using a clustering heat map (Fig. 3a). The x-axis is the clustering of samples, and the y-axis is the clustering of proteins. There were substantial differences in abundance of proteins among groups. The volcano plots provide a general overview of significantly differentially abundant proteins (Fig. 3b). The x-axis represents the log2-FC of differentially abundant proteins, and the y-axis represents the -log *P*-value as calculated by T-test. Proteins shown in black were not significantly different; red represents up-regulated proteins, and green represents down-regulated proteins. The data depict that stemazole dose affects the abundance of many proteins.

**Fig. 4:**
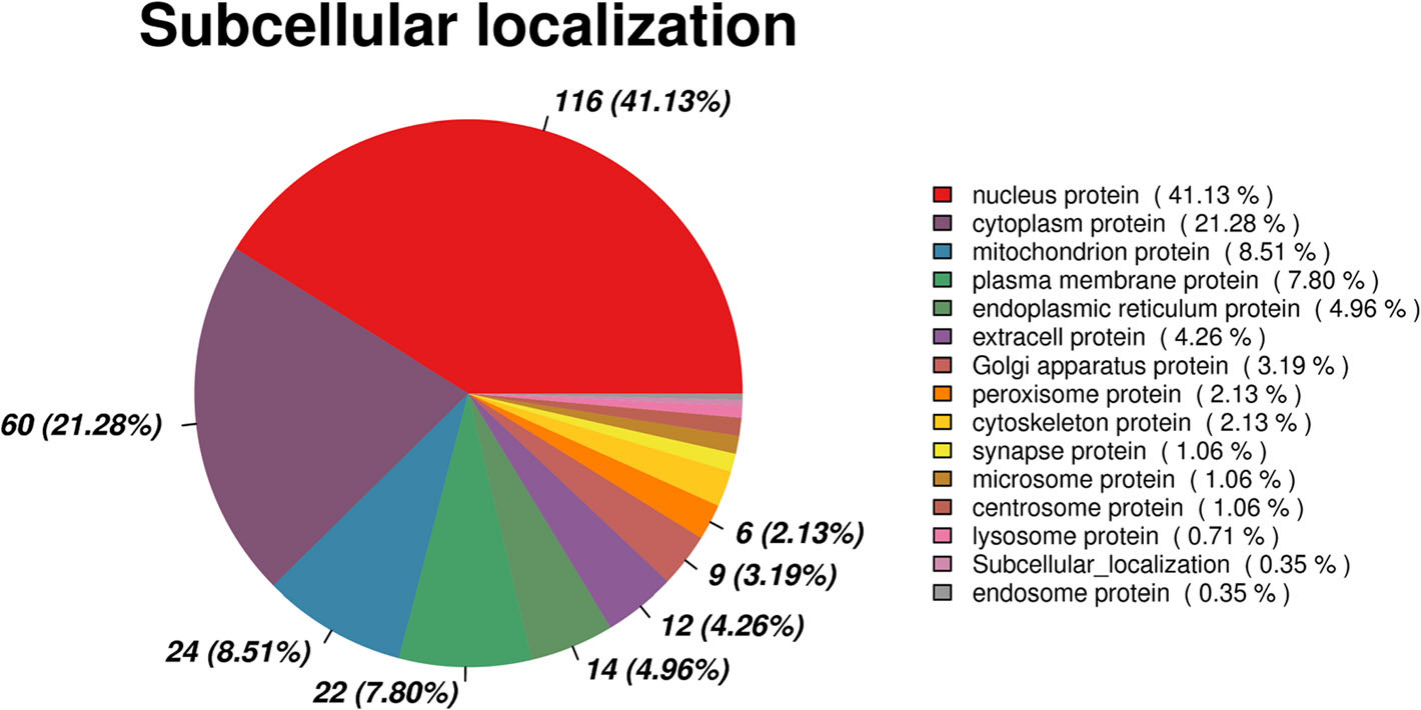
Subcellular localization analysis of differentially abundant proteins

In addition, according to the subcellular localization results, 41.13% of the differentially abundant proteins were nuclear proteins, followed by 21.28% cytoplasm proteins and 8.51% mitochondrion proteins (Fig. 4).

### GO functional annotation and enrichment analysis

**Fig. 5:**
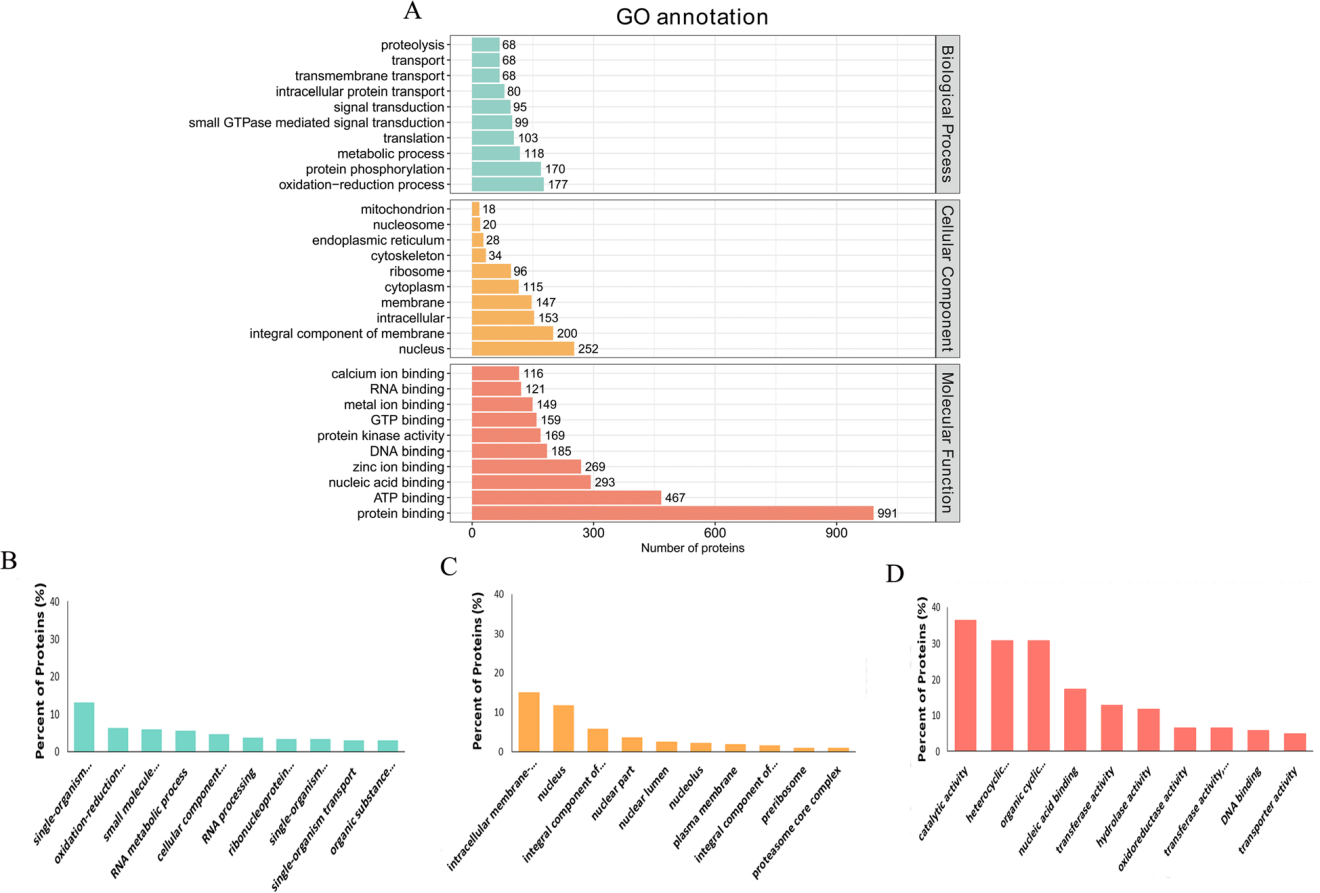
Gene Ontology (GO) functional annotation (**a**) and enrichment analysis (**b**) categorized by biological process (BPs), cellular component (CCs), and molecular function (MFs)

GO functional annotation was carried out on all the identified proteins. The GO functional annotation analysis was performed in three main annotation types, including MFs, CCs and BPs. The most representative GO terms of BPs were oxidation-reduction process and protein phosphorylation. Nucleus and integral component of membrane were the most representative GO terms of CCs. GO terms for MFs showed that most proteins were involved in protein binding and ATP binding (Fig. 5a).

To characterize the proteins affected by stemazole and to obtain an overall functional view of differentially abundant proteins, GO enrichment analysis was employed to explore which biological functions were significantly relevant. The bar plot shows the protein enrichment of differentially abundant proteins in the three categories. GO terms for MFs showed that catalytic activity was most relevant to the differences between two groups (36.39%), and heterocyclic compound binding (30.82%) and organic cyclic compound binding (30.82%) are also major annotation clusters for the differences (Fig. 5d). GO terms for BPs showed that differentially abundant proteins were involved in single-organism metabolic processes (13.11%), oxidation-reduction processes (6.23%) and small molecule metabolic processes (5.90%) (Fig. 5b). GO terms for CCs showed that differentially abundant proteins were primarily enriched in intracellular membrane-bound organelles (15.08%), the nucleus (11.80%) and integral components of membranes (5.90%) (Fig. 5c).

### KEGG pathway analysis

**Fig. 6:**
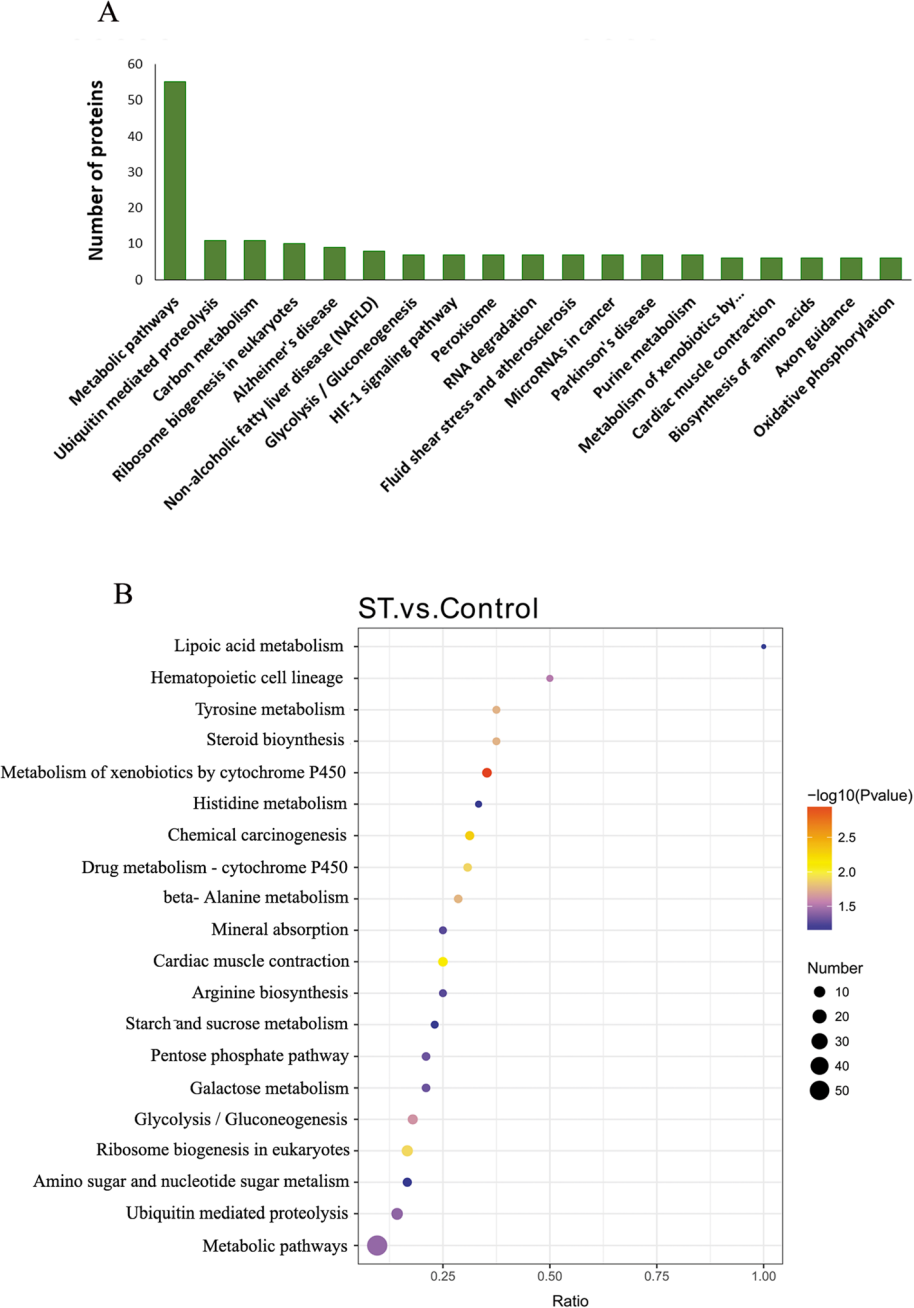
Global view of the Kyoto Encyclopedia of Genes and Genomes (KEGG) pathways in groups with or without stemazole

To further identify the biological pathways of differentially abundant proteins, KEGG pathway analysis was employed. As a result, 110 biological pathways were affected. The proteins participated in metabolic pathways, ubiquitin-mediated proteolysis and carbon metabolism pathways, with *P*-values < 0.05 (Fig. 6a) (Additional file 3: Table S2). Metabolic pathways involving 55 proteins were the most relevant, and ubiquitin-mediated proteolysis and carbon metabolism also demonstrated 11 proteins each.

According to the enrichment results, the scatter plot of the enriched KEGG pathway was drawn to obtain a more comprehensive and intuitive understanding of the overall results (Fig. 6b). The horizontal axis in the figure is the ratio of the number of differential proteins in the corresponding pathway to the total number of proteins identified by the pathway. The dot representing metabolic pathways was the largest, suggesting there were more differentially abundant proteins in this pathway. The dot of metabolism of xenobiotics by cytochrome P450 pathway was the reddest, indicating that the results were more reliable and meaningful.

### PPI network analysis

**Fig. 7:**
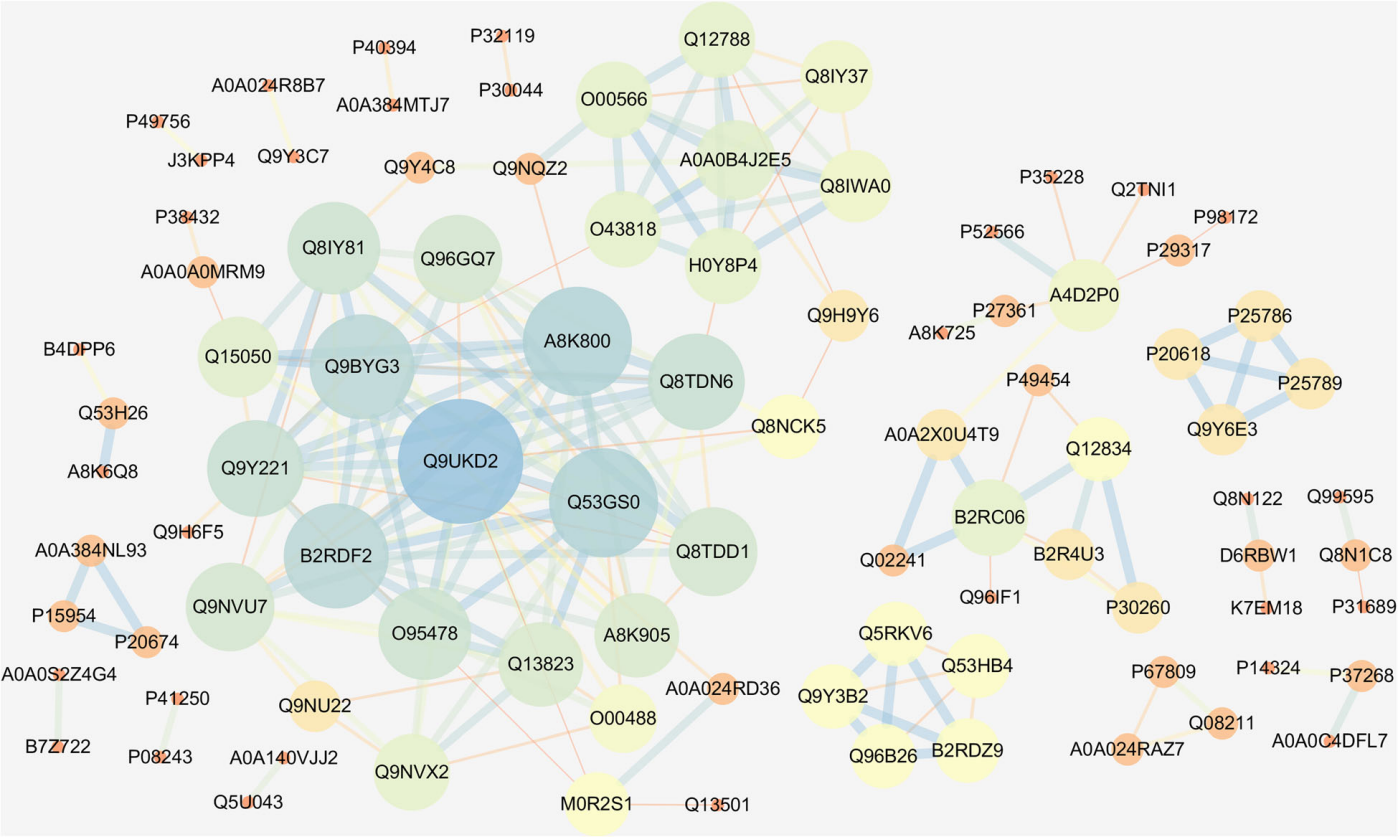
The protein-protein interaction (PPI) network results

The PPI network of the differentially abundant proteins was built with STRING database and Cytoscape software. Each interaction had a combined score that represents the reliability of the interaction between proteins. The size of the node indicates degree centrality, large size represent high degree. The color of the edge indicates the combined interaction score, dark color represent high score. In the PPI network there were 61 proteins that were discovered interacted with more than one protein (Fig. 7). Among those, Q9UKD2, Q53GS0, A8K800, Q9BYG3 and B2RDF2 were interacted with nineteen, fifteen, fifteen, fourteen and fourteen proteins, respectively.

## Discussion

The protective effects on a variety of stem cells of small molecule stemazole in extreme culture condition make it a candidate drug worth studying. To fully understand the effect of stemazole on neural stem cells, in the present study, the global protein expression profile of neural stem cells that are affected by stemazole in normal culture conditions were evaluated using a TMT-labelled proteomics approach. Proteomics analysis is a discovery-based method that provides a systematic method for the quantitative and qualitative mapping of the whole proteome and identified invariable proteins that differ in abundance between normal and stemazole groups. Detailed bioinformatics analysis was performed, including GO, KEGG pathway and PPI analyses, with the integration of multiple online servers and information databases. The obtained analysis results are reliable due to the various information sources and methods that were used.

A total of 408 differentially abundant proteins were screened. The 178 up-regulated proteins with an FC ≥ 1.2 and 230 down-regulated proteins with an FC ≤ 0.83 were selected. GO functional annotation was employed for all the identified proteins. Through annotation results, the possible functions, biological processes, signaling pathways, and other information relevant to the proteins can be known. All the identified differentially abundant proteins were functionally classified into annotation clusters. The results indicated that most differentially abundant proteins were associated with catalytic activity, including 57 up-regulated proteins and 54 down-regulated proteins.

KEGG analysis was performed by matching differentially abundant proteins with the annotated proteins in the KEGG pathway database. The frequency of differentially abundant proteins in each KEGG pathway was compared to determine their participation in the KEGG pathway. According to enrichment analysis, the number of differentially abundant proteins associated with the metabolic pathway was the largest. Comprehensively considering the *P*-values and the ratio of differential proteins in the corresponding pathway to the total proteins number, metabolism of xenobiotics by cytochrome P450 was the most relevant pathway. Cytochrome P450 enzymes include a related family of enzymes involved in the metabolism of foreign compounds [20]. This pathway contributes to the study of drug metabolism and toxicology [20, 21]. In the pathway of metabolism of xenobiotics by cytochrome P450, S-(hydroxymethyl) glutathione dehydrogenase, carbonyl reductase [NADPH] 1 (CBR1), alcohol dehydrogenase class 4 mu/sigma chain (ADH7), carbonyl reductase [NADPH] 3 (CBR3), glutathione S-transferase Mu 3 (GSTM3) and cDNA, FLJ94267, highly similar to *Homo sapiens* glutathione S-transferase omega 1 (GSTO1) mRNA, were up-regulated by stemazole. In addition, cytochrome P450 4B1 (CYP4B1) was one of the major proteins up-regulated by stemazole (Fig. 2a). This protein belongs to the mammalian CYP4 enzyme family. CYP4B1 participates in the oxidation and metabolism of structurally unrelated xenobiotics [22]. The increased abundance of CYP4B1 indicates that it is involved in the metabolism of stemazole.

Previous pharmacological mechanistic investigations of stemazole based on a network pharmacology approach identified caspase-3, caspase-8, AKT1, MAPK8, MAPK14 as the possible direct targets of stemazole and as being enriched in MAPK signalling, which is highly relevant to the occurrence and progression of neurodegenerative diseases [16]. While in this study the possible target proteins has no significant difference between the two groups. In the KEGG enrichment analysis of neural stem cells with or without stemazole, the MAPK signalling pathway was not enriched. The cell apoptosis is not activated under normal conditions. In the absence of apoptotic signals, apoptotic regulatory molecules cannot interact with each other, stemazole cannot target on these proteins. This result also proves that although stemazole possesses significant cell protection and anti-apoptosis abilities under disaggregation or starvation, it cannot play an effective role under conditions that are not extreme. The results will help us understand stemazole from another angle, and provide guidance for the application of stemazole in the future.

Cytochrome c oxidase assembly factor 3 homologue, mitochondrial (COA3) was the most up-regulated protein, as shown in Fig. 2a, and more mitochondrial proteins were down-regulated. Differential protein subcellular localization analysis results also showed that mitochondrial proteins are the proteins primarily affected. We found that stemazole leads to low abundance of several proteins involved in the NADH-ubiquinone oxidoreductase (Complex I) and cytochrome-c oxidase (Complex IV) of the ETC. The oxidative phosphorylation system consists of five enzyme complexes, which are located in the inner mitochondrial membrane. Mitochondria occupy a pivotal position in the production of aerobic ATP through oxidative phosphorylation of ADP [23, 24].

Cytochrome c oxidase subunit NDUFA4 (NDUFA4) and NADH:ubiquinone oxidoreductase MLRQ subunit homologue, isoform CRA_α (LOC56901) are two down-regulated proteins of Complex IV. Cytochrome c oxidase subunit 5A, mitochondrial (COX5A), epididymis secretory sperm binding protein, cytochrome c oxidase subunit 7A-related protein, mitochondrial (COX7A2L) and cytochrome c oxidase subunit 7C, mitochondrial (COX7C) are four down-regulated proteins of Complex I. These proteins are the enzymes in the mitochondrial electron transport chain that drive oxidative phosphorylation. There is increasing evidence that neurotherapeutics have a direct inhibitory effect on the mitochondrial respiratory electron transport chain (ETC), especially on complex I enzyme activity [25, 26]. According to our results, mitochondrial energy might be disturbed by stemazole. The lowa bundance of Complexes I and IV could cause mitochondrial dysfunction. Altogether, the results suggest that the decreased abundance of the mitochondrial proteins may be a possible cause of the overproduction of ROS and mitochondrial dysfunction, including the loss of mitochondrial membrane potential. In normal culture conditions with growth factors, low concentration of stemazole could not exhibit effect on the cells, high concentration of 4 d stemazole treatment showed cytotoxicity. So we further measured the ROS generation and mitochondrial membrane potential affected by stemazole of 4 days. Stemazole with high concentration increased ROS production, moreover, the decrease of the ratio of red and green fluorescence also demonstrated the loss of mitochondrial membrane potential (Additional file 4: Fig. S2). In normal conditions stemazole with high concentration shows cytotoxicity, it could cause overproduction of ROS and the reduction of mitochondrial membrane potential. This is worth paying attention to in future research of stemazole applications.

The finding of the possible cytotoxicity of stemazole in normal conditions defined the operational conditions for stemazole. In extreme conditions stemazole stimulates proliferation while in normal conditions stemazole cannot contact with the target apoptotic regulatory proteins cause cells apoptosis were not activated without apoptotic signals in normal condition. Stemazole does not play a protective role as it does in the extreme conditions. The different effects demonstrated under normal/extreme conditions are not contradictory, but further clarify the scope of the role of stemazole and provides a reference for more specific application of stemazole.

The discovery and reporting of cytotoxicity of high concentration stemazole is an essential part of the research cause any compound inevitably has side effects under certain conditions. As a potential drug candidate, the findings of toxicity of stemzole are necessary and valuable. As the first proteomics study of stemazole, the normal condition (with stemaole treatment, without changing other conditions) was selected first to lay a foundation for further proteome research. This study exhibits another side of this small molecular that has not been reported, which can void or minimize problems in the future research and application of stemazole.

Our results provide new insights into the protein expression profiles of neural stem cells with or without stemazole and various influences of stemazole treatment. The results of this study will contribute to a better understanding of the role of stemazole in the treatment of neurodegenerative diseases and will guide further studies of the development of stemazole as a stem cell drug.

## Conclusions

The proteomics expression profile of neural stem cells with or without stemazole was obtained. This is the first proteomics research about stemazole. A total of 408 differentially abundant proteins were screened out: 178 up-regulated proteins and 230 down-regulated proteins. The up-regulated proteins are mainly related to cytochrome P450, and some of the down-regulated are mitochondrial proteins. This study will provide a comprehensive understanding of more characteristics of stemazole and lay the foundation for future pharmaceutical study.

## Supplementary Information

**Additional file 1: Fig. S1.** Human neural stem cells viability affected by stemazole. The cells after the treatment with stemazole solution of a series of concentrations in normal culture condition were quantified by CellTiter-Glo®. Cells viability was decreased after high concentration stemazole treatment of 4 d.

**Additional file 2: Table S1.** Summary analysis results of differentially abundant proteins.

**Additional file 3: Table S2.** Summary results of KEGG enrichment analysis.

**Additional file 4: Fig. S2.** Effects of stemazole on intracellular ROS level and mitochondrial membrane potential in normal cultures. The cells were stained with a DCFH-DA probe to detect the ROS production, stemazole with high concentration could cause overproduction of ROS (a). The cells stained with JC-1 probe, red fluorescence represents the mitochondrial aggregate form of JC-1, indicating intact mitochondrial membrane potential. Green fluorescence represents the JC-1monomer, indicating dissipation of mitochondrial membrane potentials. Mitochondrial damage is indicated by a decrease in the red/green fluorescence intensity ratio. Stemazole with high concentration could cause mitochondrial damage (b).

## Data Availability

All data generated or analysed during this study are included in this article and its Additional files.

## Abbreviations

GO: Gene Ontology
KEGG: Kyoto Encyclopedia of Genes and Genomes
AD: Alzheimer’s disease
PD: Parkinson’s disease
MPTP: 1-methyl-4-phenyl-1,2,3,6-tetrahydropyridine
CASP3: caspase-3
CASP8: caspase-8
AKT1: RAC-alpha serine/threonine-protein kinase
MAPK8: mitogen-activated protein kinase 8
MAPK14: mitogen-activated protein kinase 14
TMT: tandem mass tags
FDR: false discovery rate
FC: fold change
ROS: reactive oxygen species
PPI: protein-protein interaction
CBR1: S-(hydroxymethyl) glutathione dehydrogenase, carbonyl reductase [NADPH] 1
ADH7: alcohol dehydrogenase class 4 mu/sigma chain
CBR3: carbonyl reductase [NADPH] 3
GSTM3: glutathione S-transferase Mu 3
CYP4B1: cytochrome P450 4B1
COA3: cytochrome c oxidase assembly factor 3 homologue, mitochondrial
Complex I: NADH-ubiquinone oxidoreductase
Complex IV: cytochrome-c oxidase
ETC: mitochondrial respiratory electron transport chain
NDUFA4: cytochrome c oxidase subunit NDUFA4
LOC56901: NADH:ubiquinone oxidoreductase MLRQ subunit homologue, isoform CRA_α
COX5A: cytochrome c oxidase subunit 5A, mitochondrial
COX7A2L: cytochrome c oxidase subunit 7A-related protein, mitochondrial
COX7C: cytochrome c oxidase subunit 7C, mitochondrial
FGF: fibroblast growth factor
EGF: epidermal growth factor
TEAB: triethylammonium bicarbonate
HCD: higher-energy collisional dissociation
