# Supplementary figures and images for: A quantitative proteomics analysis for small molecule Stemazole’s effect on human neural stem cells
Source: Proteome Sci. 2020 Dec 9;18:12. doi: 10.1186/s12953-020-00168-2 (PMC7724819; doi:10.1186/s12953-020-00168-2)

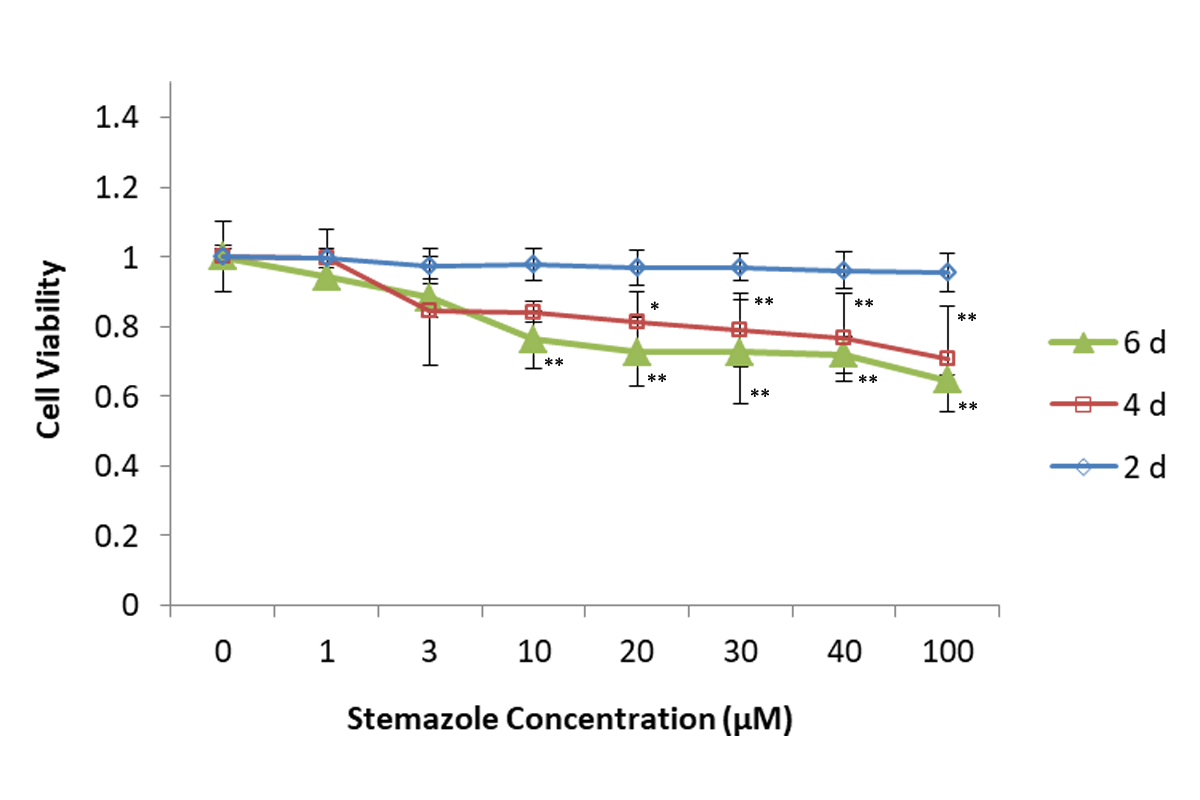

Supplement: Supplementary file 1 — Additional file 1: Fig. S1. Human neural stem cells viability affected by stemazole. The cells after the treatment with stemazole solution of a series of concentrations in normal culture condition were quantified by CellTiter-Glo®. Cells viability was decreased after high concentration stemazole treatment of 4 d. [file 12953_2020_168_MOESM1_ESM.tif]

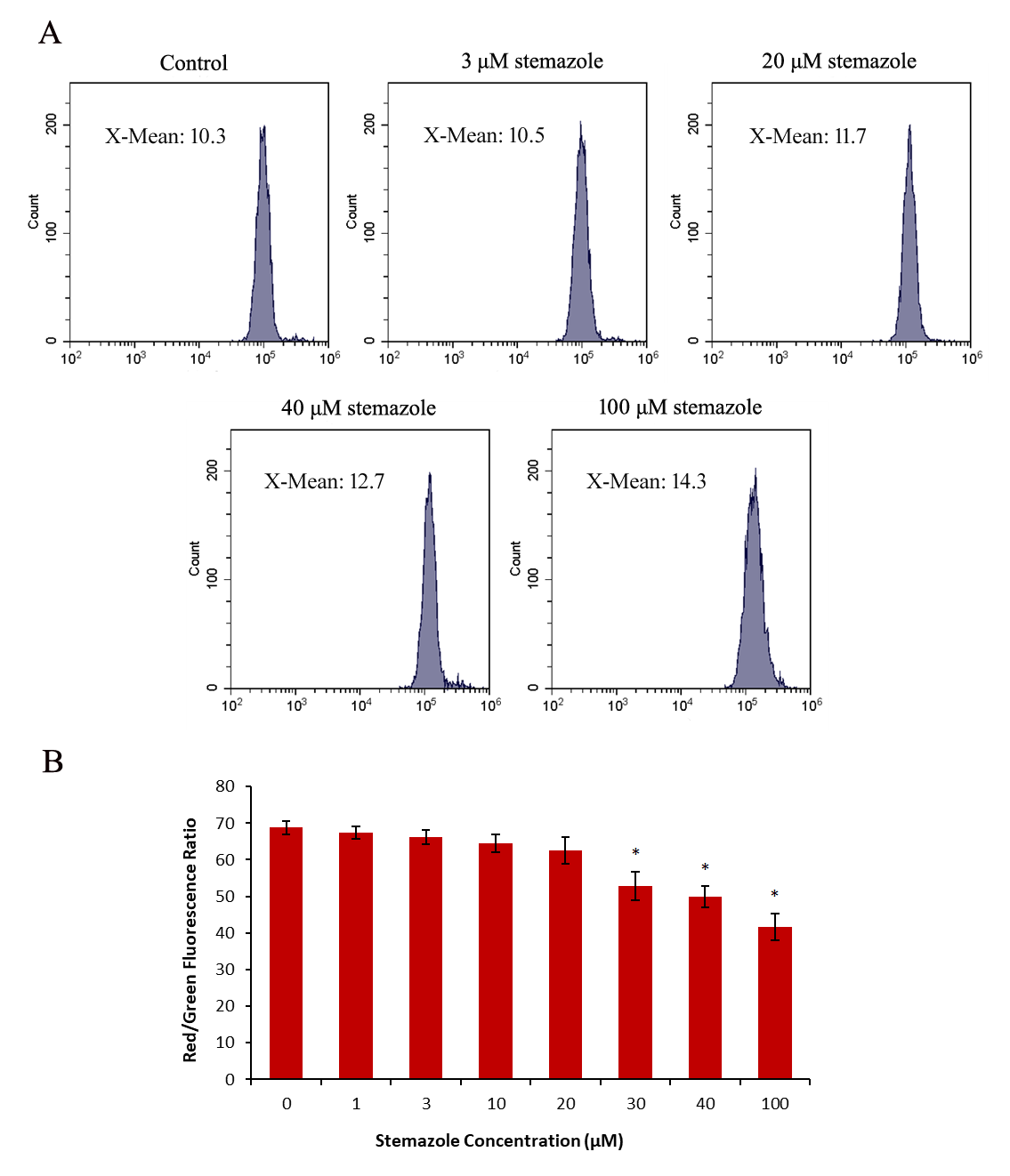

Supplement: Supplementary file 4 — Additional file 4: Fig. S2. Effects of stemazole on intracellular ROS level and mitochondrial membrane potential in normal cultures. The cells were stained with a DCFH-DA probe to detect the ROS production, stemazole with high concentration could cause overproduction of ROS (a). The cells stained with JC-1 probe, red fluorescence represents the mitochondrial aggregate form of JC-1, indicating intact mitochondrial membrane potential. Green fluorescence represents the JC-1monomer, indicating dissipation of mitochondrial membrane potentials. Mitochondrial damage is indicated by a decrease in the red/green fluorescence intensity ratio. Stemazole with high concentration could cause mitochondrial damage (b). [file 12953_2020_168_MOESM4_ESM.tif]
